# Reduced protection of RIPK3-deficient mice against influenza by matrix protein 2 ectodomain targeted active and passive vaccination strategies

**DOI:** 10.1038/s41419-022-04710-2

**Published:** 2022-03-29

**Authors:** Teodora Oltean, Lorena Itati Ibanez, Tatyana Divert, Tine Ysenbaert, Hannelore Van Eeckhoutte, Vera Goossens, Michael Schotsaert, Ken Bracke, Bert Schepens, Jonathan Maelfait, Nozomi Takahashi, Xavier Saelens, Peter Vandenabeele

**Affiliations:** 1 VIB-UGent, Center for Inflammation Research (IRC), Ghent, Belgium; 2 Department of Biomedical Molecular Biology (DBMB), Ghent, Belgium; 3 VIB-UGent Center for Medical Biotechnology, VIB, Ghent, Belgium; 4 Department of Biochemistry and Microbiology, Ghent University, Ghent, Belgium; 5 Laboratory for Translational Research in Obstructive Pulmonary Diseases, Dept of Respiratory Medicine, Ghent University Hospital, Ghent, Belgium; 6 VIB Screening Core & UGent Expertise Centre for Bioassay Development and Screening (C-BIOS), Ghent, Belgium

**Keywords:** Infection, Viral infection

## Abstract

RIPK3 partially protects against disease caused by influenza A virus (IAV) infection in the mouse model. Here, we compared the immune protection of active vaccination with a universal influenza A vaccine candidate based on the matrix protein 2 ectodomain (M2e) and of passive immunization with anti-M2e IgG antibodies in wild type and *Ripk3*^−/−^ mice. We observed that the protection against IAV after active vaccination with M2e viral antigen is lost in *Ripk3*^*−/−*^ mice. Interestingly, M2e-specific serum IgG levels induced by M2e vaccination were not significantly different between wild type and *Ripk3*^*−/−*^ vaccinated mice demonstrating that the at least the humoral immune response was not affected by the absence of RIPK3 during active vaccination. Moreover, following IAV challenge, lungs of M2e vaccinated *Ripk3*^*−*/−^ mice revealed a decreased number of immune cell infiltrates and an increased accumulation of dead cells, suggesting that phagocytosis could be reduced in *Ripk3*^−/−^ mice. However, neither efferocytosis nor antibody-dependent phagocytosis were affected in macrophages isolated from *Ripk3*^*−*/−^ mice. Likewise following IAV infection of *Ripk3*^−/−^ mice, active vaccination and infection resulted in decreased presence of CD8+ T-cells in the lung. However, it is unclear whether this reflects a deficiency in vaccination or an inability following infection. Finally, passively transferred anti-M2e monoclonal antibodies at higher dose than littermate wild type mice completely protected *Ripk3*^*−/−*^ mice against an otherwise lethal IAV infection, demonstrating that the increased sensitivity of *Ripk3*^−/−^ mice could be overcome by increased antibodies. Therefore we conclude that passive immunization strategies with monoclonal antibody could be useful for individuals with reduced IAV vaccine efficacy or increased IAV sensitivity, such as may be expected in patients treated with future anti-inflammatory therapeutics for chronic inflammatory diseases such as RIPK inhibitors.

## Graphical abstract

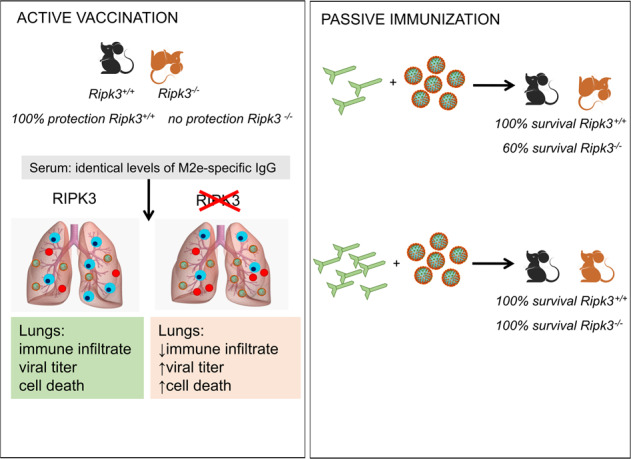

## Introduction

Seasonal human influenza viruses cause acute respiratory infections which affect the entire population and kill up to 650,000 people worldwide each year, and are responsible for substantial public health burden and economic cost [1]. Currently, yearly vaccination is considered the most effective measure to prevent or reduce disease caused by influenza A and B viruses. Seasonal influenza vaccines are mostly based on inactivated influenza viruses and their composition is reevaluated yearly for each hemisphere to follow the antigenic drift of the circulating influenza viruses. Influenza vaccines based on the highly conserved extracellular domain of matrix protein 2 (M2e) of influenza A, have been proposed as possible alternatives for currently licensed influenza vaccines [2, 3]. Immunization of laboratory mice with M2e displayed on a virus-like particle (VLP) protect against a potentially lethal influenza A virus (IAV) challenge [4]. This protection can be transferred by serum, requires a functional Fcγ receptor compartment and is mediated by antibody-dependent cellular phagocytosis [4, 5]. Phase I studies with M2e-based vaccine candidates have been completed, which suggested that such vaccine candidates are safe and immunogenic in healthy volunteers (e.g., NCT00819013) [6]. Whether M2e-based prophylactic vaccination strategies prevent or reduce disease caused by IAV infection in humans remains to be determined. A controlled IAV challenge study in healthy volunteers revealed that a dose of 40 mg/kg of a human anti-M2e IgG1 monoclonal antibody was associated with a significant reduction in the total influenza symptom score compared to the placebo treated group [7]. To date, preclinical and clinical M2e-based influenza A vaccine development efforts are continuously being explored [8].

Cell death signaling pathways contribute to the innate immune defense against infectious diseases. One key player in some of these pathways is Receptor-Interacting serine/threonine-Protein Kinase 3 (RIPK3) [9]. Indeed, RIPK3 is involved in the protection against IAV infection by several mechanisms [10–14]. RIPK3 kinase activity, for example, is implicated in TNF-and ZBP1-mediated necroptosis, while as a scaffold it is implicated in apoptosis upon IAV infection [14]. Beyond its involvement in the protection against IAV infection, the contribution of RIPK3 to vaccine-induced immune protection conferred by active vaccination strategies has not been addressed. In this study, we compared the immunogenicity of active vaccination with M2e-VLPs and the protective potential of passive transfer of M2e-specific IgG monoclonal antibodies in wild type and *Ripk3*-deficient mice against an IAV challenge.

## Results

### RIPK3 is required for protection against IAV following active vaccination with M2e-VLP

**Fig. 1:**
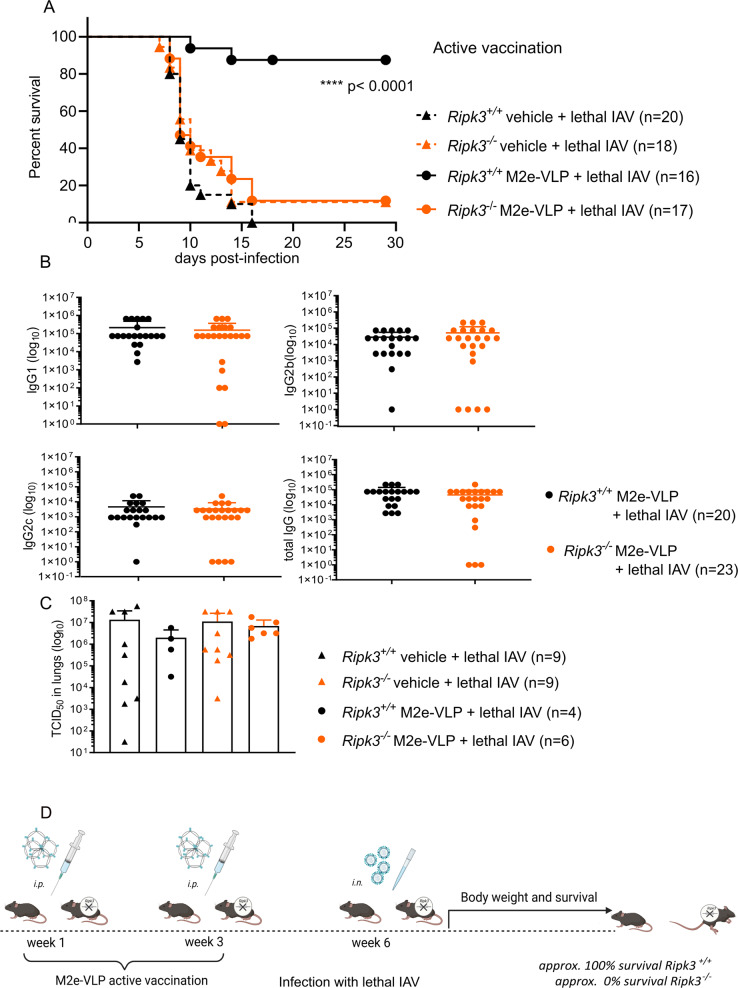
Ripk3-deficient mice are not protected against IAV lethal infection following active vaccination with M2e-VLP despite similar serum levels of M2e-specific antibodies and viral clearance. **A** Active vaccination with M2e-VLP particles was administered with Alhydrogel® adjuvant intraperitoneally. Age-matched *Ripk3*^*+/+*^ and *Ripk3*^*−/−*^ mice received either 5 µg/mouse M2e-VLP with Alhydrogel^®^ adjuvant vaccination or just the Alhydrogel® adjuvant dissolved in PBS (vehicle) 3 weeks and 6 weeks before infection. Lethal challenge was done with the mouse-adapted influenza X47 with either 2 × LD_50_ (virus batch 1) or 0.5 × LD_50_ (virus batch 2). The experiment was repeated four times independently and data were pooled. Survival curves were plotted for indicated groups and evaluated statistically according to Kaplan–Meier. A log-rank test verified significant differences between *Ripk3*^*+/+*^ and *Ripk3*^*−/−*^ mice post-vaccination; ****p-value< 0.0001 (GraphPad Prism 8). Similar numbers of female and male mice were used comparing both genotypes (19 females, 3 males *Ripk3*^*+/+*^ versus 21 females, 6 males *Ripk3*^*−/−*^). The body weight readout of this experiment is provided in Supplementary Fig. 2A. **B** RIPK3-deficient mice effectively produce specific antibodies against M2e: Age-matched *Ripk3*^*+/+*^ and *Ripk3*^*−/−*^ mice were primed (6 weeks before infection) then immunized (3 weeks before infection) 10 μg M2e-VLP with Alhydrogel® adjuvant or received PBS with Alhydrogel^®^ adjuvant (vehicle). Serum samples were prepared two weeks after each immunization. Titers of IgG1 IgG2a IgG2b and total IgG against M2e were measured after the immunization were determined by ELISA. The legend shows the M2e-specific antibody titers obtained for individual mice (dots) of each group from three independent experiments. **C** Virus clearance from the lungs post vaccination is independent from RIPK3-deficiency: On day 6 post-infection, lungs were harvested and lung homogenates were assessed for viral titers by TCID_50_. Data for pooled lung homogenates from different mice (two independent experiments) of the same group are shown. The means for TCID_50_ are shown for each group as indicated in the legend. Error bars represent SD. **D** Overview of the active vaccination strategy and the results obtained in *Ripk3*^*+/+*^ and *Ripk3*^*−/−*^ mice.

To evaluate if active vaccination remains effective in a host that is susceptible to IAV infection, we immunized *Ripk3*^*−*^^/−^ mice with a broad-spectrum influenza A vaccine candidate based on M2e, and subsequently challenged these mice with a lethal dose of IAV (Fig. 1). The *Ripk3*^*−/−*^ mice and their *Ripk3*^*+/+*^ littermates were primed and immunized with the M2e-VLP vaccines or with phosphate-buffered saline (PBS), as a negative control. As expected at this high IAV challenge dose, there is no difference between unvaccinated *Ripk3*^*−/−*^ versus *Ripk3*^*+/+*^ mice [14]. Almost all *Ripk3*^*−/−*^ mice, though vaccinated died soon after infection (Fig. 1A and Supplementary Fig. 2A), while most *Ripk3*^*+/+*^ littermates survived. This indicates that RIPK3 not only is involved in the protection against IAV infection [14, 15], but is also crucial for the efficacy of vaccination against IAV.

Both the innate and acquired immune responses are required for an effective immune response post active vaccination [16]. Therefore, we checked whether the absence of protection of RIPK3-deficient mice following vaccination would be due to impaired production of specific anti-M2e antibodies (Fig. 1B). Remarkably, sera taken from *Ripk3*^*−/−*^ mice isolated 2 weeks after the boost with M2e-VLP revealed similar levels of M2e-specific IgG antibodies as the *Ripk3*^*+/+*^ littermates. This finding suggests that besides the antibody production post immunization, other mechanisms contribute to effective vaccination which would be lacking in RIPK3-deficient mice. Active vaccination with M2e-VLP decreases viral titers in the lungs upon IAV challenge [4]. Since RIPK3 is important for viral protection we examined whether viral titers would be different in vaccinated *Ripk3*-deficient and—proficient animals. At day 6 post-infection, we measured viral titers in the lungs of non-vaccinated and vaccinated mice. We observed that in non-vaccinated mice, the viral loads were similar between the *Ripk3*^*+/+*^ and the *Ripk3*^*−/−*^ mice. However, both in *Ripk3*^*+/+*^ and *Ripk3*^*−/−*^ mice vaccination reduces the viral load, however, this tendency is less outspoken in *Ripk3*^*−/−*^ (from mean 1.1 × 10^7^ to mean 6.9 × 10^6^, or a reduction of about 40%) compared to the *Ripk3*^*+/+*^ mice (from mean 1.34 × 10^7^ to mean 2 × 10^6^, or a reduction of about 90%) (Fig. 1C). These data suggest that the lack of protection after vaccination in *Ripk3*^*−/−*^ mice might be due to increased virus loads in the lungs as a consequence of decreased vaccination efficacy or increased IAV sensitivity and virus propagation.

### IAV-associated inflammation is reduced in vaccinated *Ripk3*^*−/−*^ mice

**Fig. 2:**
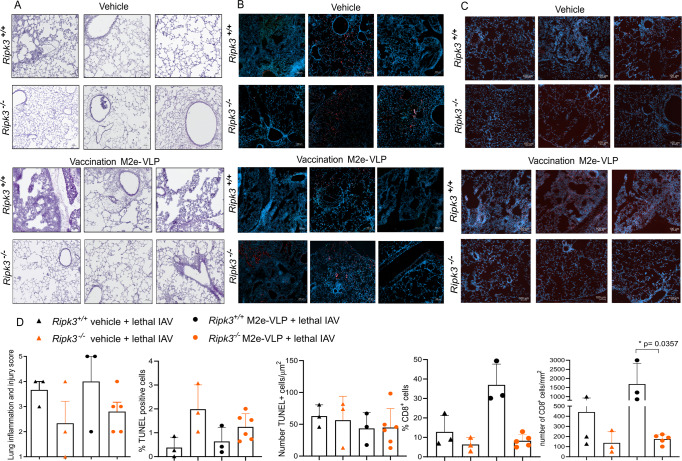
Immune cells infiltration in the lungs is required for protection against IAV lethal infection. **A** Vaccinated and non-vaccinated *Ripk3*^*+/+*^ and *Ripk3*^*−/−*^ mice challenged with lethal IAV dose challenge (2 × LD_50_; virus batch 1) were sacrificed at 6 days post-infection and their lungs were collected and stained with hematoxylin-eosin. Representative images of individual mice revealed less inflammation in the vaccinated *Ripk3*^*−/−*^ mice compared to their *Ripk3*^*+/+*^ control. **B** Vaccinated and non-vaccinated *Ripk3*^*+/+*^ and *Ripk3*^*−/−*^ mice challenged with lethal IAV were sacrificed at 6 days post-infection and their lungs were collected. **C** Representative images displaying TUNEL positivity (red) and cells nuclei (blue) are shown in (**B**) and CD8 positivity (red) and cell nuclei (blue) is shown in (**C**). A tendency to accumulate more dying-cells in the lungs of *Ripk3*^*−/−*^ mice compared to their *Ripk3*^*+/+*^ controls is observed. **D** The extent of lung inflammation and injury score was blindly scored (0 - no inflammatory cell infiltration; normal alveolar septa; 1 - mild peribronchial/perivascular inflammatory cell infiltration in parts of the lung; normal alveolar septa; 2 - moderate peribronchial/perivascular inflammatory cell infiltration in parts of the lung; mild thickening of alveolar septa; 3 - severe alveolar and interstitial inflammatory cell infiltration in parts of the lung; moderate thickening of alveolar septa; 4 - very severe alveolar and interstitial inflammatory cell infiltration in parts of the lung; severe thickening of alveolar septa; 5 - very severe alveolar and interstitial inflammatory cell infiltration throughout the lung; severe thickening of alveolar septa). TUNEL positivity and for CD8 positivity was scored with a software 0.2.3. and QuPath software 0.3.0. respectively. Graphics and statistical analysis were done with GraphPad Prism 8. Comparisons were done with Mann–Whitney test in GraphPad Prism 8.

In order to understand the underlying phenomena explaining the difference in vaccination efficiency in *Ripk3*^*−/−*^ and *Ripk3*^*+/+*^ mice, we determined immune cells infiltration in the lungs following vaccination and infection. Indeed immune cell infiltration is required for protection against IAV lethal infection [17]. We observed that the lung inflammation score of non-vaccinated and vaccinated *Ripk3*^*−/−*^ showed a similar tendency for reduced inflammation by histology and injury score (Fig. 2A), less immune infiltrates (Supplementary Fig. 1A) and a higher amount of dead cell as detected by TUNEL staining compared to littermate controls (Fig. 2B). However, quantification of these parameters did not reveal significance because only discrete areas are affected (Fig. 2D and Supplementary Fig. 1B). It was previously shown that IAV-specific CD8+ T cell numbers were significantly diminished upon infection in *Ripk3*^*−/−*^ mice compared to littermate controls [10]. These CD8+ T are important for the support and efficient effector immune response. Here, we also observed that in the lung the number of CD8+ cells was more pronouncedly decreased in the vaccinated *Ripk3*^*−/−*^ mice compared to the vaccinated *Ripk3*^*+/+*^ littermates (Fig. 2C, D). All these observations suggest that the *Ripk3*^*−/−*^ mice may have an impaired innate immune response against IAV leading to reduced protection or enhanced sensitivity.

**Fig. 3:**
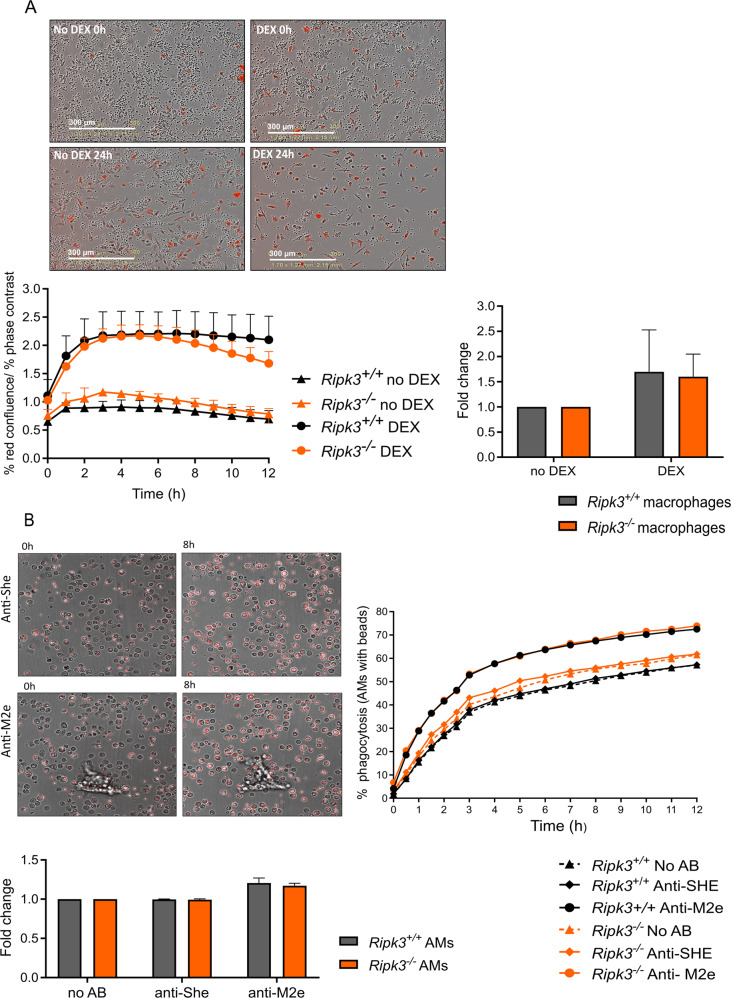
RIPK3-deficient macrophages are competent to perform efferocytosis and antibody-dependent cellular phagocytosis. **A** Primary peritoneal macrophages isolated from *Ripk3*^*+/+*^ and *Ripk3*^*−/−*^ mice were co-incubated in 1:5 ratio with apoptotic (dexamethasone-killed, DEX) or non-apoptotic (no DEX) thymocytes stained with pH-sensitive dye, CypHER 5E. Live cell imaging was performed with IncuCyte^®^ S3 (Sartorius). Representative images of macrophages which engulfed DEX-treated or no DEX-treated thymocytes become positive for CypHER 5E (red) can be seen. The difference between *Ripk3*^*+/+*^ and *Ripk3*^*−/−*^ at 8 h of co-incubation is shown as fold change relative to the no DEX control (three independent experiments). **B** M2e-coated fluorescent polystyrene beads were incubated in 2.5:1 ratio with primary alveolar macrophages isolated form *Ripk3*^*+/+*^ or *Ripk3*^*−/−*^ mice in the absence or presence of 0.1 μg/mL of isotype control antibody, Anti-SHE or an anti-M2e mouse IgG2a monoclonal antibody (Mab65) specific antibody, Anti-M2e. Live cell imaging was performed with Operetta high content imaging system and analysis was done with Harmony software. The difference between *Ripk3*^*+/+*^ and *Ripk3*^*−/−*^ at 8 h of co-incubation is shown as fold increase in antibody-mediated phagocytosis relative to no antibody control (three independent experiments). Significance was determined using one-way ANOVA with Tukey correction (GraphPad Prism 8). Error bars represent SEM.

One possibility is that RIPK3 might be implicated in immune cell infiltration and removal of dead cell corpses reflecting a possible deficit in the capacity of executing efferocytosis and antibody-dependent cellular phagocytosis due to reduced recruitment of phagocytes or reduced phagocytic efficacy, or a combination of both. However, *Ripk3*-deficient peritoneal and alveolar macrophages are as competent as littermate control to perform efferocytosis and antibody-dependent phagocytosis, respectively. Indeed, we found that RIPK3 deficiency does not affect the capacity of peritoneal macrophages to engulf dexamethasone-treated apoptotic thymocytes (Fig. 3A). Since Fc receptors and alveolar macrophages have been demonstrated to be crucial for the protection against lethal IAV infection by passive transfer of anti-M2e IgG [5], we also examined a possible impairment of the process of antibody-dependent cellular phagocytosis (ADCP). To this end, we developed a method in which M2e-coated polystyrene beads mimicking infected cells were incubated with primary alveolar macrophages isolated from *Ripk3*^*+/+*^ or *Ripk3*^*−/−*^ mice in the presence or absence of M2e-specific antibodies. We observed that *Ripk3* deficiency does not affect the capacity of alveolar macrophages to engulf M2e-coated beads with or without antibodies, demonstrating that RIPK3 is also dispensable for ADCP (Fig. 3B). Therefore we conclude that very likely the reduced recruitment of immune cells combined with the enhanced sensitivity of RIPK3-deficient mice for IAV infection [10–14] may explain the observed reduced survival of *Ripk3*^*−/−*^ mice following vaccination.

### Increased doses of passive immunization with Anti-M2e monoclonal antibodies can completely protect *Ripk3*-deficient mice

**Fig. 4:**
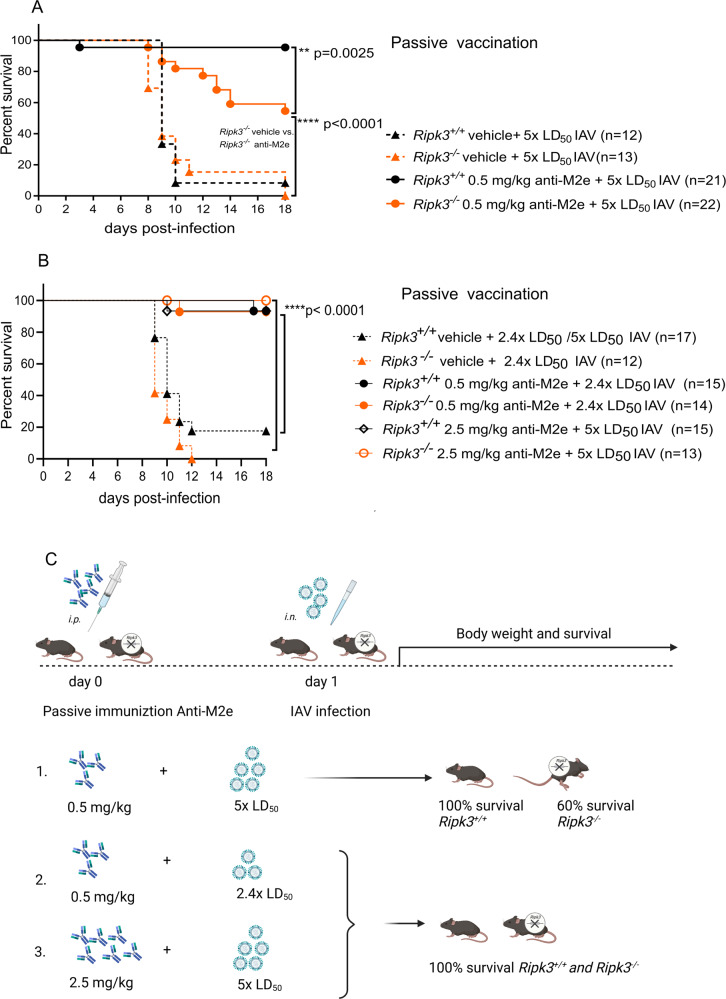
Increased doses of passive immunization with Anti-M2e monoclonal antibodies can completely protect *Ripk3*-deficient mice. **A** The administration of a standard dose Anti-M2e (0.5 mg/kg) and challenge with lethal IAV (5 × LD_50_; viral batch 3) (four independent experiments, total number of mice indicated between brackets). The passive immunization with monoclonal Anti-M2e was done i.p. one day before the i.n. infection with IAV. Survival was monitored daily for up to 18 days post-challenge. Survival curves were plotted for indicated groups and evaluated statistically according to Kaplan–Meier (GraphPad Prism 8). Similar numbers of female and male mice were used comparing both genotypes (18 females, 15 males *Ripk3*^*+/+*^ versus 18 females, 17 males *Ripk3*^*−/−*^). The body weight readout of this experiment is provided in Supplementary Fig. 2B. Cox regression did not reveal any significant difference (*p* = 0.517) in survival between female and male mice. **B** The combination of passive immunization with a standard Anti-M2e dose (0.5 mg/kg) and a decreased (but still lethal) IAV dose (2.4 × LD_50_; viral batch 3) was performed three times (total number of mice indicated between brackets). The combination of passive immunization with an increased dose of Anti-M2e (2.5 mg/kg) and lethal IAV dose (5 × LD_50_; viral batch 3) in three independent experiments (total number of mice indicated in the legends). The passive immunization with monoclonal Anti-M2e was done i.p. one day before the i.n. infection with IAV. Survival was monitored daily for up to 18 days post-challenge. Survival curves were plotted for indicated groups and evaluated statistically according to Kaplan–Meier (GraphPad Prism 8). The body weight readout of this experiment is provided in Supplementary Fig. 2C. **C** Scheme of passive immunization and main conclusions. Overview of the results in terms or survival of *Ripk3*^*+/+*^ and *Ripk3*^*−/−*^ mice are shown in approximative percentages based on the results in panels (**A** and **B**). Similar numbers of female and male mice were used comparing both genotypes (27 females, 14 males *Ripk3*^*+/+*^ versus 26 females, 13 males *Ripk3*^*−/−*^).

Passive transfer of anti-M2e monoclonal antibodies protects mice against IAV infection [18]. We wondered whether the increased sensitivity to IAV lethality in *Ripk3*-deficient mice could be rescued by passive transfer of anti-M2e monoclonal antibodies providing a protective alternative for inefficient active immunization in *Ripk3*^*−/−*^ mice. In case of a lethal dose of IAV infection (5× LD_50_), wild type littermates were completely protected by passive transfer of a moderate dose of anti-M2e antibodies (10 µg/20 g or 0.5 mg/kg), while almost half of the *Ripk3*^*−/−*^ mice succumbed (Fig. 4A and Supplementary Fig. 2B). Cox regression did not reveal any significant difference (*p* = 0.517) between female and male mice exhibiting partial protection in *Ripk3*^*−/−*^ during passive vaccination. However, when higher amounts of anti-M2e monoclonal antibodies (50 µg/20 g or 2.5 mg/kg) were used in the passive transfer in combination with the same viral dose for challenge (5× LD_50_), then all *Ripk3*^*−/−*^ mice were also completely protected (Fig. 4B and Supplementary Fig. 2C). If mice were infected with a lower viral dose (here 2.4× LD_50_) in combination with the standard dose of anti-M2e (10 µg/20 g or 0.5 mg/kg) we also reached complete protection in *Ripk3*^*−/−*^ mice (Fig. 4B and Supplementary Fig. 2C). Altogether these passive vaccination results in conditions of decreased anti-viral protection show a balance between the capacity of passively transfer M2e antibodies to cope with infection and the dose of infection (Fig. 4C). This demonstrates that a deficiency in an antiviral response gene such as *Ripk3* could be compensated by sufficient levels of passively administered monoclonal antibodies.

## Discussion

In this study, we examined whether a state of fairly enhanced sensitivity to IAV infection in *Ripk3*^*−/−*^ mice [14], would affect the efficacy of active immunization with M2e viral antigen or passive immunization by transfer of anti-M2e monoclonal antibodies. Since RIPK3, both as a kinase and a scaffold protein, is implicated in many cellular processes such as necroptosis, apoptosis, inflammasome activation, and pyroptosis induction, its loss may be associated with a reduced induction of an anti-viral state in an infected host. This could be a model for populations at risk for severe IAV infection such as elderly, children, pregnant women, and immunodeficient patients [19, 20]. To date, a potential correlation between RIPK3 single nucleotide polymorphism (SNPs) and an increased susceptibility to IAV in human hosts has not been investigated. Additional investigation needs to be done to explore this possibility. Given the development of RIPK1 and RIPK3 inhibitors as anti-inflammatory treatments for chronic inflammatory diseases [21–23], our work reveals an important caveat. Future patients receiving such RIPK inhibitors may be immunocompromised and could benefit from monoclonal antibody therapy overcoming any deficits in influenza immunity. We and others showed that, at least in mice, RIPK3 is an important mediator of protection against IAV infection [10, 14, 15, 24–26]. However, we have reported previously that this sensitizing effect of RIPK3 deficiency is only limited to a certain level of viral challenge. Indeed, morbidity and lethality after a high IAV challenge *Ripk3*^*−/−*^ mice were comparable to those in their *Ripk3*^*+/+*^ littermates [14].

Interestingly, active immunization of *Ripk3*^*−/−*^ mice with recombinant M2e-VLPs could raise equal titers of M2e-specific serum IgG antibodies as compared to immunization of *Ripk3*^*+/+*^ mice, but this apparently does not result in a protective effect against a lethal influenza challenge. The full ability of *Ripk3*^*−/−*^ mice to produce antibodies against M2e suggests that other factors than M2e-specific antibodies contribute to an efficient antibody-mediated protection. We found that non-vaccinated and vaccinated *Ripk3*^*−/−*^ mice display reduced immune cells infiltration in the lungs and accumulate more dying cells post infection compared to their wild type littermates. Conceptually, these phenomena could be due to decreased ADCP and efferocytosis. However, our experiments did not reveal a difference in these processes in peritoneal and alveolar-derived macrophages from *Ripk3*^*+/+*^ littermates and *Ripk3*^*−/−*^ mice. Since the *Ripk3*^*−/−*^ mice are able to produce normal levels of M2e-specific antibodies following M2e-VLP immunization, humoral immune responses following active immunization are not impaired in *Ripk3-*deficient mice. However, despite these equal levels of protective antibodies, a differential response was observed following viral challenge between *Ripk3*^*−/−*^ mice and *Ripk3*^*+/+*^ littermates. The former showed a tendency for increased viral loads following vaccination, decreased inflammation score, reduced infiltration of CD8+ immune cells, and accumulation of dead cell corpses in the lungs, suggesting that the innate arm is compromised in *Ripk3*-deficient mice. We do not exclude that other immune cells important in the IAV clearance such as neutrophils and NK cells could also be reduced in the vaccinated *Ripk3*^*−/−*^ mice contributing to their inability to efficiently respond to active vaccination.

Although most of the differences in viral load, inflammation, and cell death parameters in the lung between the vaccinated *Ripk3*^*+/+*^ *and Ripk3*^*−/−*^ mice only showed a tendency and were not significant, the combination of these three parameters can have a crucial impact on the lethal phenotype observed in *Ripk3*^*−/−*^ mice despite vaccination. Also, we cannot exclude that there might be a difference in the induction of M2e-specific T cells [25]. Altogether, our work supports two potential, non-exclusive scenarios for the enhanced sensitivity of *Ripk3*^*−/−*^ mice in the active vaccination model. One possibility is that non-humoral arms of vaccine-induced immunity are impaired under RIPK3 deficiency leading to reduced numbers of CD8+ T-cells. The other possibility reflects a cell-autonomous role of RIPK3 in controlling IAV infection up to mid-range IAV doses. In order to examine whether the immunocompromised *Ripk3*-deficient mice could still be protected by antibodies, we delivered anti-M2e monoclonal antibodies prior to IAV infection. The transfer of high dose of anti-M2e monoclonal antibodies protects the *Ripk3*^*−/−*^ mice from a lethal IAV infection [18] while a moderate dose does not. Passive transfer with anti-M2e monoclonal antibodies was shown to limit viral load in the infected lungs which can modulate the susceptibility of the *Ripk3*^*−/−*^ mice to the IAV making them less susceptible to the same dose of infection [14, 26]^,^. It is clear from our experiments that modulation of the viral dose in the lungs is crucial for the survival of *Ripk3*^*−/−*^ mice [14]. Protective host strategies against IAV infection consist in the capacity to rapidly recognize and eliminate the virus or to quickly regain fitness by reducing the negative impact of infection [27]. It seems that *Ripk3*^*−/−*^ mice are not able to reduce the pathogen burden when it reaches a certain threshold, and eventually succumb to the infection [14]. Interestingly, active vaccination helps them to produce antibodies, but not to sufficiently to reduce viral titers, therefore *Ripk3*^*−/−*^ mice, are not protected by this vaccination. However, when passive immunization is used at a high dose, this could decrease viral burden and rescue the *Ripk3*^*−/−*^ mice from lethal IAV infection.

The reduced infiltration of immune cells and CD8+ cells observed in *Ripk3*^*−/−*^ mice may also be explained, at least partly, by reduced vascular permeability. Indeed, *Ripk3*^*−/−*^ mice have reduced endothelial permeability affecting tumor migration into the lung using a B16 melanoma model [28]. It is therefore conceivable that a similar endothelial mechanism may also contribute to reduced diffusion of antibodies in lung tissue and BAL fluid, in line with the reduced efficacy of humoral protection despite identical titers following active vaccination and with the higher amount of monoclonal antibodies required for protection following passive vaccination. To examine such a role of RIPK3 in the endothelial compartment during active and passive vaccination, one would need to perform experiments in endothelium-specific *Ripk3*^*−/−*^ mice.

This finding provides a clinically relevant option for patients at risk which may not respond to active vaccination and argues that in antiviral compromised organisms the passive vaccination may bypass the affected innate immune system. For example, one study on adults hospitalized with acute respiratory illness during the 2017–18 influenza season in the USA showed that the deduced influenza vaccine effectiveness in hospitalized immunocompromised patients was as low as 5% compared to 41% in the immunocompetent individuals [28]. Furthermore, emerging data show that monoclonal antibodies are promising candidates for the treatment of IAV infections in the future [29, 30], whenever vaccines are not effective. Some of these antibodies are currently being evaluated in clinical trials [31]. These monoclonal antibody-based anti-IAV strategies may also be required to cope with the actual COVID-19 pandemic and the continuous occurrence of variants.

## Material and methods

### Mice

*Ripk3*^*−/−*^ and littermate controls *Ripk3*^*+/+*^ were kindly provided by Vishva Dixit (Genentech, San Francisco). The *Ripk3*^*−/−*^ animals were congenic to the C57BL/6N background. All mice were housed in individually ventilated cages in a conventional animal house. 7–13 weeks-old mice were used in all experiments. All animal experiments were performed under conditions specified by law (European Directive and Belgian Royal Decree of November 14, 1993) and reviewed and approved by the Institutional Ethics Committee on Experimental Animals (EC2016-17).

### Active vaccination with M2e-VLP and virus challenge

Age-matched *Ripk3*^*+/+*^ and *Ripk3*^*−/−*^ mice were intraperitoneally injected two times with 5 µg of purified M2e-VLP in the absence or presence of Alhydrogel^®^ adjuvant (Brenntag Biosector Specification, total volume, 200 µl). The M2e-VLP 1965, expressed by and purified from recombinant *E. coli* cells, was used for active vaccination and comprises 1–162 amino acids of HBc and they are able to entrap bacterial RNA [31]. Control mice received a vehicle containing Alhydrogel^®^ adjuvant in PBS, pH 7.4. The two injections were given at 3-week intervals. Three weeks after the last immunization, mice were challenged with a lethal dose of mouse-adapted of X47 influenza virus. Depending on the viral batch preparation the challenge following active vaccination was with a lethal dose of either 2 × LD_50_ [1 × LD_50_ corresponding to approximately 30 tissue culture infectious dose 50 (TCID_50_)] (virus batch 1) or 0.5 × LD_50_ (1 × LD_50_ corresponds with 80 plaque-forming units or pfu) (virus batch 2). The virus was administered intranasally in a total volume of 50 µl to mice anesthetized by ketamine (44 mg/kg) and xylazine (5 mg/kg). Mice were either monitored for survival and weight loss over a period of 18 days. We used the following 4 scores of clinical symptoms: 0 = no visible signs of disease; 1 = slight ruffling of fur; 2 = ruffled fur, reduced mobility; 3 = ruffled fur, reduced mobility, rapid breathing; 4 = ruffled fur, minimal mobility, huddled appearance, rapid and/or labored breathing indicative of pneumonia and body temperature below 32 °C. For the combination of body weight loss by 30% and a clinical score 4 the mice were considered moribund and euthanized by CO_2_ asphyxiation or cervical dislocation (EC2016-17).

### Serum preparation and analysis of the production of antibodies against M2e

Blood samples were obtained from every mouse, before immunization, after the first boost, and after the second boost. Serum was prepared and the presence of M2e-specific antibodies was determined by ELISA, as described previously [4].

### Passive transfer of Anti-M2e monoclonal antibodies and virus challenge

Purified Anti-M2e IgG monoclonal antibody (clone mAb 65) [18] or isotype control were i.p. injected at 0.5 mg/kg or 2.5 mg/kg as indicated in the figure legends (200 µl/mouse) in naive mice. After 24 h, the mice were anesthetized with a mixture of ketamine (44 mg/kg) and xylazine (5 mg/kg) and challenged by intranasal administration of 50 μl of different doses of mouse-adapted X47 (H3N2) IAV, as indicated in the figure legends. For passive immunization experiments challenges were performed with lethal doses of 2.4 × LD_50_ or 5 × LD_50_ of a viral batch in which 1 × LD_50_ corresponds to 175 pfu (virus batch 3). Mortality and body weight loss were monitored for up to 30 days after challenge.

### TCID_50_ assay

TCID_50_ assays were used to determine the amount of infectious virus in lung homogenates of each condition. MDCK cells cultured in Dulbecco’s modified Eagle’s medium (DMEM) supplemented with 10% FCS, nonessential amino acids, 2 mM L-glutamine, and 0.4 mM Na-pyruvate were seeded in 96-well plates to reach confluence overnight at 37 °C in 5% CO_2_. The cells were washed in serum-free medium and incubated with 10-fold dilutions of virus samples containing 1 μg/ml of TPCK-treated trypsin (Sigma). After 6 days, the presence of virus in each well was determined by agglutination of chicken erythrocytes. TCID_50_ values were calculated based on the Reed and Muench method [32].

### Lung histology

Lungs were collected from indicated mice sacrificed on day 6 post-infection. Tissues were covered in cryo-embedding media, kept in liquid nitrogen until completely submerged, then stored at −80 °C until ready for sectioning. 4 mm sections were cut with a cryotome and stained with haematoxylin and eosin. For immunofluorescence, sections were fixed in 4% paraformaldehyde for 1 h at RT, washed with PBS, then incubated in “permeabilisation solution” containing 0.05% TX-100 and 0.1% sodium citrate for 2 min on ice (4 °C). Cell death was analyzed with an in situ cell death detection kit (TMR-red, Roche) after antigen retrieval either alone or followed by staining with anti-CD45 (AB 10558 Abcam) and Goat anti-Rabbit IgG DyLight488 (ThermoFicher ref: 35553). Anti-CD8a (AB 217344, Abcam) was used 1/100 combined with a secondary Goat anti-Rabbit IgG AF568. Hoechst 33342 was used to stain nuclei. Brightfield and fluorescence microscopy was performed using ZEISS Axio Scan.Z1. Samples were analyzed with Zen 3.2. blue edition (Zeiss) and quantified with QuPath software 0.2.3.

### Macrophage isolation

Resident peritoneal macrophages were obtained by flushing the peritoneal cavity of mice with 10 ml of cold PBS containing 5% FCS. Collected cells were spun down and resuspended in RPMI 1640 medium supplemented with 10% FCS, 10 mM Na-pyruvate, L-glutamine, penicilin/strepomycin (100 U/100 Ag/ml), HEPES, β-mercaptoethanol, and 100 mM non-essential amino acids. The cells were plated at a concentration of 4.5 × 10^5^ cells per well in a 24-well plate and maintained at 37 °C in a 5% CO_2_ humidified atmosphere. Floating cells were washed away the next day and remaining peritoneal macrophages were used 2 days after isolation. Alveolar macrophages were collected from mice were anesthetized via intraperitoneal injection of Nembutal (pentobarbital; 125 mg/kg in PBS; Lundbeck, Valby, Denmark). A small incision was made in the trachea to put a lavage cannula in the trachea. Lungs were lavaged three times with 1 ml of HBSS with 0.05 mM EDTA (Sigma-Aldrich) and the bronchoalveolar lavage fluid (BALF) was kept on ice. Collected cells were centrifuged, resuspended in RPMI 1640 medium supplemented with 10% FCS, 10 mM Na-pyruvate, L-glutamine, penicillin/streptomycin (100 U/100 Ag/ml), HEPES, β-mercaptoethanol, and seeded at 1 × 10^5^ cells per well in a 96-well plate. Cells were allowed to adhere at 37 °C in a 5% CO_2_ humidified atmosphere for 2 h before phagocytosis assays.

### Beads preparation and ADCP

Amine-modified polystyrene beads (Polysciences) were pre-activated with 8% (vol/vol) glutaraldehyde for 4 h at room temperature. Beads were conjugated with 10 µg/ml of M2e peptide and 0.2 mg/ml of Alexa Fluor N-hydroxy-succinimide ester dyes (Life Technologies) on a rotating wheel overnight at 4 °C. After quenching in PBS containing 0.5 M glycine for 2 h, beads were used for ADCP assays. Beads were incubated in 2.5:1 ratio with freshly isolated alveolar macrophages in the absence or presence of 0.1 μg/ml of isotype control antibody, anti-SHE (IgG2a isotype control MAbs directed against the small hydrophobic protein of human respiratory syncytial virus) [33] or M2e-specific antibody [34, 35]. Live cell imaging was performed with Operetta high content imaging system and analysis was done with Harmony software (PerkinElmer).

### Efferocytosis assay

Thymocytes were isolated from 4 to 6-week-old mice and induced to undergo apoptosis with 20 µM dexamethasone for 4 h at 37 °C in 5% CO_2_ incubator. Thymocytes were then stained with CypHer5E (GE Healthcare), counted, and added to adherent peritoneal macrophages at a macrophage: apoptotic target ratio of 1:5. Live cell imaging was performed and analyzed with IncuCyte^®^ S3 (Sartorius).

## Supplementary information

Legends Suppl. Figures

Suppl. Figure 1

Suppl. Figure 2

Checklist form

Author contribution form

## Data Availability

The datasets used and/or analyzed during the current study are available from the corresponding author on reasonable request.
