# Supplementary material for: Reduced protection of RIPK3-deficient mice against influenza by matrix protein 2 ectodomain targeted active and passive vaccination strategies
Source: Cell Death Dis. 2022 Mar 29;13(3):280. doi: 10.1038/s41419-022-04710-2 (PMC8961492; doi:10.1038/s41419-022-04710-2)
Supplement: Supplementary file 1 — Legends Suppl. Figures [file 41419_2022_4710_MOESM1_ESM.docx]

**Supplementary Figures**

**Supplementary Figure 1. Lung cells, but not myeloid cells die in the lungs of immunized and non-immunized mice. A**) Vaccinated and non-vaccinated *Ripk3^+/+^* and *Ripk3^-/-^* mice challenged lethal IAV dose (2 x LD_50_; virus batch 1) were sacrificed at 6 days post-infection and their lungs were collected. Representative pictures showing lung regions from 3 different mice (from left to right): TUNEL positive cells (red) and myeloid cells stained with Anti-CD45 (green). **B**) Quantification of TUNEL+, CD45+ and TUNEL-CD45+ cells was done with a script generated for the QuPath 0.2.3 software. A very low amount of CD45+ myeloid cells are TUNEL+. More TUNEL+ can be seen in the vaccinated *Ripk3^-/-^* mice compared to *Ripk3^+/+^*.

**Supplementary Figure 2. Body weight loss of *Ripk3^-/-^* mice and their *Ripk3^+/+^* littermates receiving active or passive immunization against IAV. A**) Active vaccination with M2e-VLP particles was administered with Alhydrogel® adjuvant intraperitoneally. Age-matched *Ripk3^+/+^* and *Ripk3^-/-^* mice received either 5 µg/mouse M2e-VLP with Alhydrogel®adjuvant vaccination or just the Alhydrogel® adjuvant dissolved in PBS (vehicle) 3 weeks and 6 weeks before infection. The follow up of the body weight was done after lethal IAV challenge with 2 x LD_50_ (virus batch 1) or 0.5 x LD_50_ (virus batch 2) for up to 22 days post-infection. Body weight curves were generated with GraphPad Prism 8 and are shown as mean ± SD. **B**) The passive immunization with monoclonal Anti-M2e was done i.p. one day before the i.n. infection with IAV. Body weight was monitored daily for up to 18 days post-challenge. Body weight curves of mice receiving passive transfer of standard dose Anti-M2e (0.5 mg/kg) and challenge with lethal IAV (5 x LD_50_; viral batch 3). **C**) Body weight curves of mice receiving either a combination of the standard Anti-M2e dose (0.5 mg/kg) and a decreased (but lethal) IAV dose (2.4 x LD_50_; viral batch 3) and an increased dose of Anti-M2e (2.5 mg/kg) combined with the lethal IAV dose (5 x LD_50_; viral batch 3).
