# Supplementary figures and images for: Reduced protection of RIPK3-deficient mice against influenza by matrix protein 2 ectodomain targeted active and passive vaccination strategies
Source: Cell Death Dis. 2022 Mar 29;13(3):280. doi: 10.1038/s41419-022-04710-2 (PMC8961492; doi:10.1038/s41419-022-04710-2)

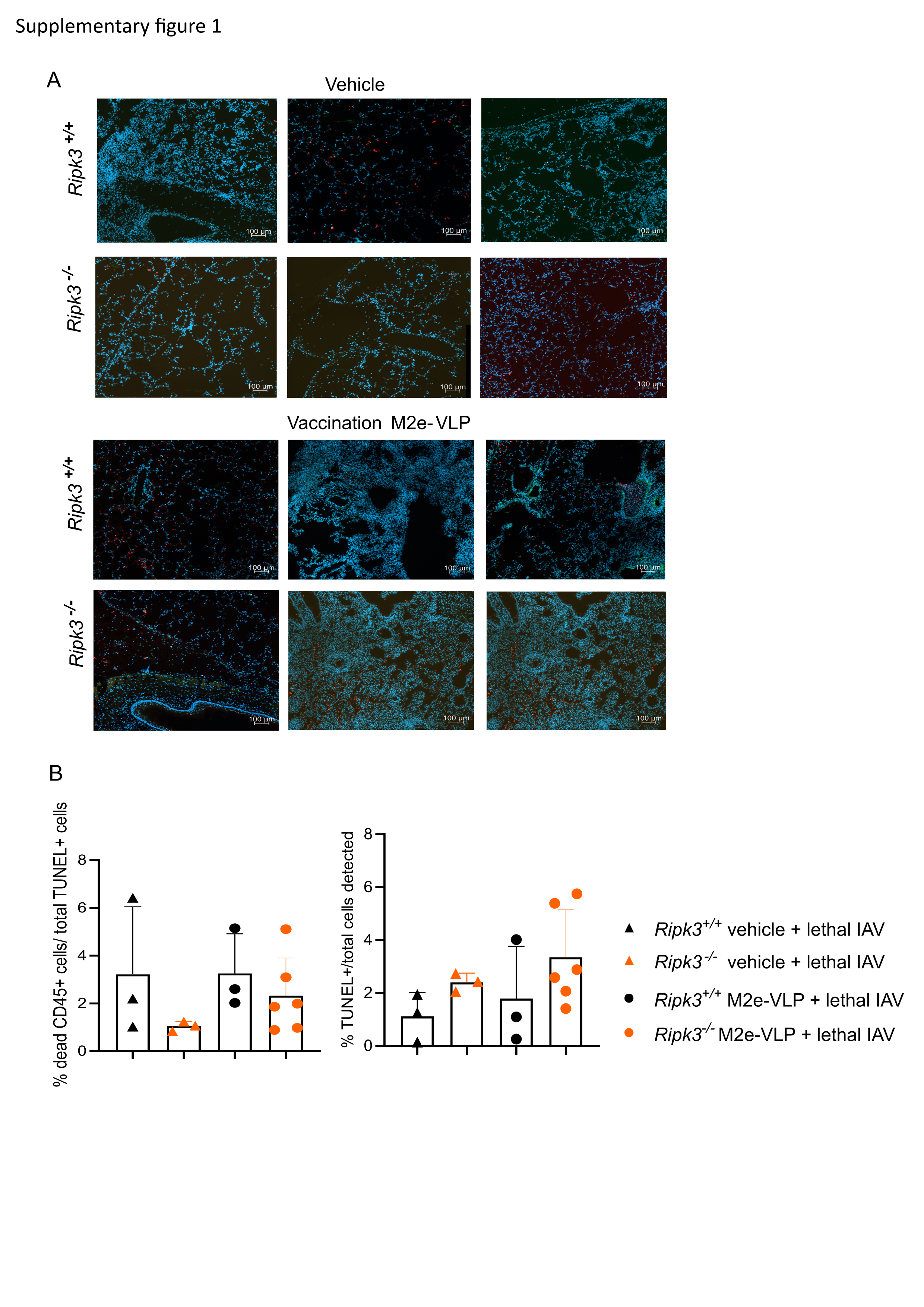

Supplement: Supplementary file 2 — Suppl. Figure 1 [file 41419_2022_4710_MOESM2_ESM.png]

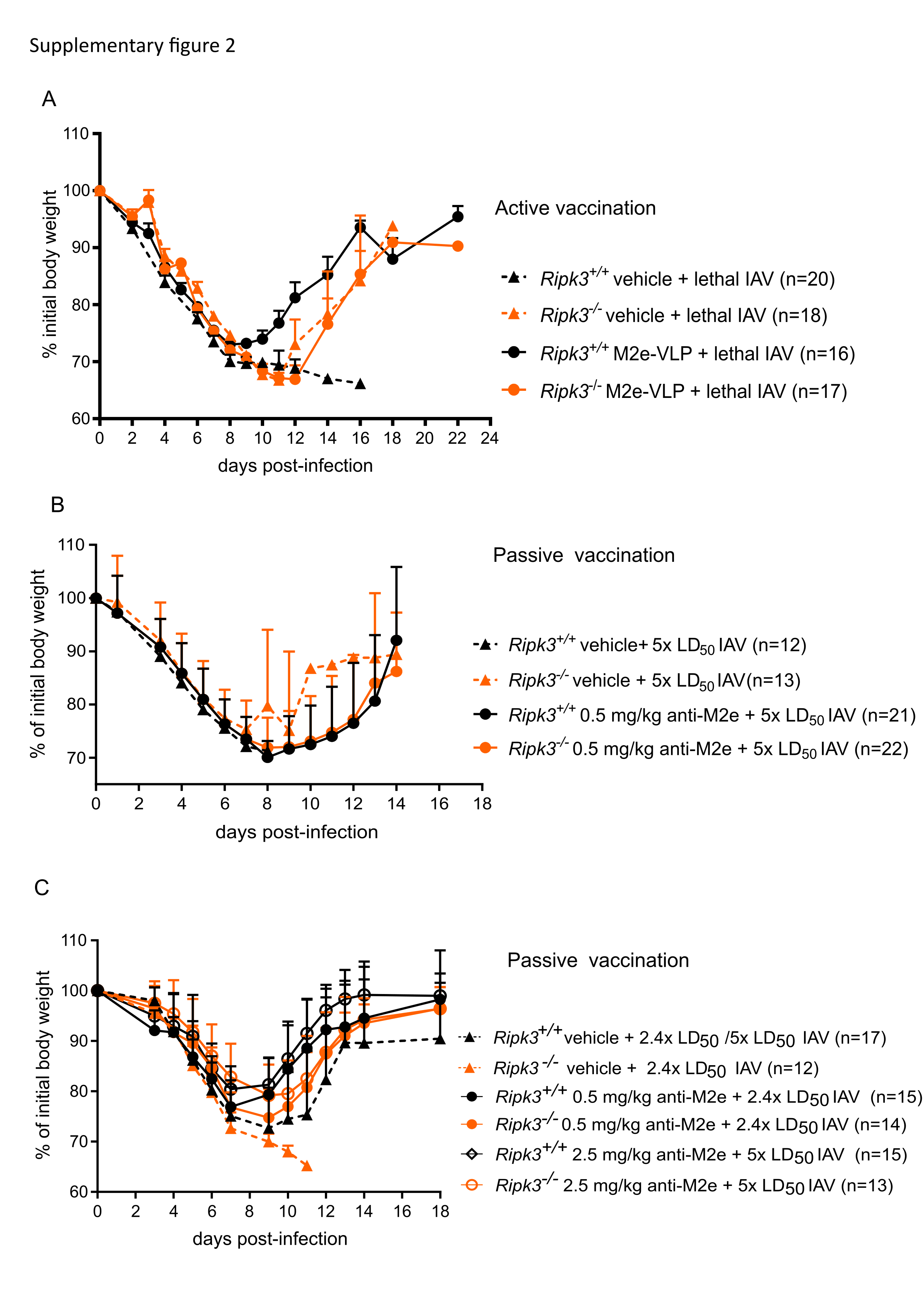

Supplement: Supplementary file 3 — Suppl. Figure 2 [file 41419_2022_4710_MOESM3_ESM.png]
